# Dissociable responses to punishment in distinct striatal regions during reversal learning

**DOI:** 10.1016/j.neuroimage.2010.03.036

**Published:** 2010-07-15

**Authors:** Oliver J. Robinson, Michael J. Frank, Barbara J. Sahakian, Roshan Cools

**Affiliations:** a Department of Psychiatry and Behavioural and Clinical Neuroscience Institute, University of Cambridge, Cambridge, Addenbrooke's Hospital, P. O. Box 189, Level E4, Hills Road, Cambridge, CB2 2QQ, UK; b Section on Neuroimaging in Mood and Anxiety Disorders, National Institute of Mental Health, National Institutes of Health, Bethesda, MD, USA; c Dept of Cognitive & Linguistic Sciences, Dept of Psychology, Brown Institute for Brain Science, Dept of Psychiatry and Human Behavior, Brown University, USA; d Donders Institute for Brain, Cognition and Behaviour, Centre for Cognitive Neuroimaging, Department of Psychiatry, Radboud University Nijmegen Medical Centre, Nijmegen, The Netherlands

## Abstract

Adaptive behavior depends on the ability to flexibly alter our choices in response to changes in reward and punishment contingencies. One brain region frequently implicated in such behavior is the striatum. However, this region is functionally diverse and there are a number of apparent inconsistencies across previous studies. For instance, how can significant BOLD responses in the ventral striatum during punishment-based reversal learning be reconciled with the frequently demonstrated role of the ventral striatum in reward processing? Here we attempt to address this question by separately examining BOLD responses during reversal learning driven by reward and during reversal learning driven by punishment. We demonstrate simultaneous valence-specific and valence-nonspecific signals in the striatum, with the posterior dorsal striatum responding only to unexpected reward, and the anterior ventral striatum responding to both unexpected punishment as well as unexpected reward. These data help to reconcile conflicting findings from previous studies by showing that distinct regions of the striatum exhibit dissociable responses to punishment during reversal learning.

## Introduction

Adequate adaptation to the environment requires the anticipation of biologically relevant events by learning signals of their occurrence. In our constantly changing environment, however, this learning must be highly flexible. The experimental model that has been used most frequently to study the neurobiological mechanisms of this learning is the *reversal learning* paradigm in which subjects reverse choices when they unexpectedly receive punishment.

Lesions of the striatum impair reversal learning in animals (Divac et al., 1967, Taghzouti et al., 1985, Annett et al., 1989, Stern & Passingham, 1996, Clarke et al., 2008) and increased striatal BOLD signal is seen in humans during the punishment events that lead to reversal (Cools et al., 2002, Dodds et al., 2008). This signal is disrupted by dopamine-enhancing drugs in both Parkinson's patients (PD) and healthy volunteers (Cools et al., 2007, Dodds et al., 2008). However, the mechanism underlying punishment-related signal in the striatum during reversal learning is unclear.

The role of dopamine and the striatum in *punishment* is receiving an increasing amount of attention (Frank, 2005, Seymour et al., 2007b, Delgado et al., 2008, Cohen & Frank, 2009). Frank (2005) has proposed that dopamine-induced impairment in punishment learning reflects disruption of striatal processing associated with punishment and subsequent suppression (NOGO) of actions associated with aversive outcomes (Frank et al., 2004, Shen et al., 2008, Cohen & Frank, 2009). The striatum has been linked to aversive processing in animals (Ikemoto & Panksepp, 1999, Horvitz, 2000, Schoenbaum et al., 2003), fMRI studies have demonstrated aversive prediction errors within the striatum (Jensen et al., 2003, Jensen et al., 2007, Seymour et al., 2005, Seymour et al., 2007a, Delgado et al., 2008) and the effect of dopaminergic medication in PD on punishment learning is comparable to the effect on reward learning (Frank et al., 2004, Cools et al., 2006, Bodi et al., 2009).

However, it is possible that striatal activity during reversal learning reflects *reward* processing (Cools et al., 2007). fMRI studies have reported greater-than-average activity in the striatum for reward prediction errors alongside less-than-average activity for punishment prediction errors (Kim et al., 2006, Pessiglione et al., 2006, Yacubian et al., 2006, Tom et al., 2007). Many midbrain dopamine neurons exclusively exhibit reward-signed coding (Schultz, 2002, Ungless, 2004 but see Brischoux et al., 2009, Matsumoto & Hikosaka, 2009) and baseline firing rates of dopamine neurons are relatively low, leaving little opportunity for decreases in firing to code negatively signed prediction errors (Daw et al., 2002, Bayer & Glimcher, 2005; but see Bayer et al., 2007). Disruption of striatal activity by dopaminergic drugs may represent abolition of activity related to reward anticipation or dopamine-induced potentiation of reward-related activity during the baseline correct rewarded responses (Cools et al., 2007). Here we address the question whether striatal activity during reversal learning represents reward- or punishment-related processing.

## Materials and methods

To disentangle these hypotheses, we assessed BOLD responses in the striatum during two intermixed types of reversal learning. Unlike previous instrumental reversal learning tasks, the received outcome in the present task did not depend on the subject's choice. Thus subjects could not work to gain positive and to avoid negative outcomes. Rather subjects were required to learn to predict the outcome (reward or punishment) for a series of stimuli. This set-up enabled us to assess striatal responses during reversals that were driven either by reward or by punishment, but which were otherwise matched exactly in terms of sensory and motor requirements. Thus one type of reversal required the detection and updating of outcome predictions in response to *unexpected punishment*, whereas the other type of reversal required the detection and updating of outcome predictions in response to *unexpected reward*. Unlike previous studies with instrumental contingencies, the reversal events in this experiment did not load highly on reward anticipation because they were not by definition followed by reward.

## Subjects

Procedures were approved by the Cambridge Research Ethics Committee (07/Q0108/28) and were in accord with the Helsinki Declaration of 1975. Sixteen right-handed subjects (mean age 26; standard error 1; five female) participated in this study at the Wolfson Brain Imaging Centre (WBIC) in Cambridge, UK. They were screened for psychiatric and neurological disorders, gave written informed consent, and were compensated for participation. Exclusion criteria were all contraindications for fMRI scanning; cardiac, hepatic, renal, pulmonary, neurological, psychiatric or gastrointestinal disorders, and medication/drug use. All subjects successfully completed the study. Blocks that quit automatically (due to 10 consecutive errors, see below) or blocks that were interrupted by the subject (i.e. to go to the toilet) were excluded (between one and three blocks for four subjects).

## Behavioral measures

### Task description

The paradigm was adapted from a previously developed paradigm and programmed using E-PRIME (Psychological Software Tools, Inc., Learning 2002; Research and Development Center, University of Pittsburgh, Pennsylvania).

**Fig. 1:**
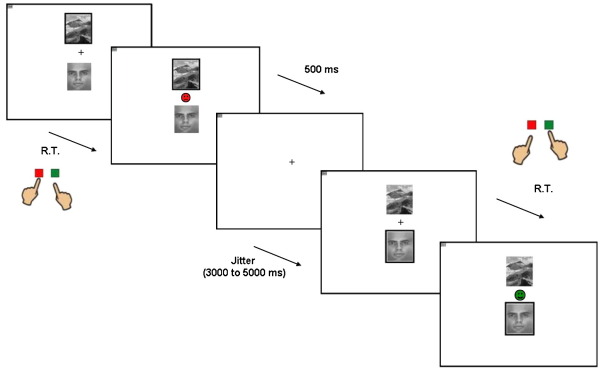
Example task-sequence of two trials. Abbreviation: R.T. = reaction time. Subjects see two stimuli, one of which is highlighted with a black box (top left). They then have to predict whether the highlighted stimulus will be followed by reward or punishment (pressing a green or red button respectively). This is then followed by the actual outcome associated with the particular stimulus. Reversals are signaled by unexpected outcomes.

On each trial subjects were presented two vertically adjacent stimuli, one scene and one face (location randomized) on a screen viewed by means of a mirror attached to the head coil in the fMRI scanner. One of these two (face/scene) stimuli was associated with reward, whereas the other was associated with punishment. Subjects were required to learn these deterministic stimulus-outcome associations by trial and error. However, unlike standard reversal paradigms, the present task did not require the subjects to choose between the two stimuli. Instead subjects were instructed to predict whether a stimulus that was highlighted with a thick black border (randomized from trial to trial) would lead to reward or to punishment. They indicated their outcome prediction for the highlighted stimulus by pressing, with the index or middle finger of their dominant (right) hand, one of two buttons on a button box placed upon their abdomen. They pressed one button for reward and the other for punishment. The outcome-response mappings were counterbalanced between subjects (left or right reward; eight in each group). Subjects had up to 1500 ms in which to make their response. As soon as they responded, the outcome was presented for 500 ms in the center of the screen (between the two stimuli). Reward consisted of a green smiley face and punishment consisted of a red sad face. If they failed to make a response, “too late!” was displayed instead of feedback. After the outcome, the screen was cleared (except for a fixation cross) for an RT-dependent interval, so that the inter-stimulus-interval was jittered modestly between 3000 and 5000 ms. Specifically, the screen was cleared for 1500 ms minus the RT and remained clear until the next scanner pulse occurred. The pulse was followed by a jittered wait of either 500 or 1500 ms before the next two stimuli appeared on the screen (Fig. 1).

After acquisition of preset learning criteria (see below) the stimulus-outcome contingencies reversed multiple times. Such reversals of contingencies were signaled to subjects either by an unexpected reward presented after the previously punished stimulus was highlighted, or by an unexpected punishment presented after the previously rewarded stimulus was highlighted. Unexpected reward and unexpected punishment events were interspersed within blocks.

The stimulus that was highlighted after the unexpected outcome was randomly selected such that it was the same as the one paired, on the previous trial, with the unexpected outcome on 50% of trials (stimulus repetition). On the other 50% of trials, the highlighted stimulus was different from the one paired with the unexpected outcome (stimulus alternations). Subjects were required to reverse their predictions in both cases. However, after an unexpected outcome, stimulus-repetitions required response alternation, whereas stimulus-alternation required response repetition. Randomizing stimulus-repetition and stimulus-alternation prevented the potential confound of response anticipation after unexpected outcomes. Furthermore, it ensured that reward anticipation and response requirements such as response inhibition and response activation were minimized, and matched between unexpected reward and punishment trial types.

During the scan session subjects completed six experimental blocks. Each experimental block consisted of one acquisition stage and a variable number of reversal stages. The task proceeded from one stage to the next following a specific number of consecutive correct trials as determined by a preset learning criterion. This criterion varied between stages (4, 5 or 6 correct responses) to prevent predictability of reversals. The average number of reversal stages per experimental block was 18 (see Results), although the block terminated automatically after completion of 150 trials (10 min), such that each subject performed 900 trials (six blocks) per experimental session (approximately 70 min including breaks). The task also terminated after 10 consecutive incorrect trials in order to avoid scanning blocks in which subjects were not performing the task correctly (e.g. due to forgetting of outcome-response mappings).

Each subject performed a practice block prior to entering the fMRI scanner to familiarize them with the task. This practice task was identical to the main task with the exception that the stimuli were presented on a pace-blade tablet computer and responses were made on a computer keyboard.

### Behavioral analysis

Errors were broken down into A) switch errors (*incorrect responses on the trial after an unexpected outcome*) and B) perseverative errors (*the number of consecutive errors after the unexpected outcome [excluding the switch trial] before a correct response was made*) and were stratified by the unexpected outcome preceding the trial, as well as by the newly associated (to be predicted) outcome. We also examined C) repetition errors (*switch trials on which subjects should have repeated responses but alternated*), D) alternation errors (*switch trials on which subjects should have switched responses but repeated*) and E) non-switch errors (*stratified by the associated [to be predicted] outcome*).

Although subjects had no control over the outcomes, it is possible that they nevertheless exhibited valence-dependent response biases to aversive or appetitive predictions and outcomes that are immaterial to the delivery of the outcomes (Dayan & Huys, 2008, Dayan and Seymour, 2008). We considered two possible types of biases:

1) *Win-stay, lose-shift strategy:* subjects might exhibit approach in response to the receipt of rewards and withdrawal in response to the receipt of punishments. In our task, such responses might be expressed in terms of a tendency to repeat the same response after a reward, while alternating the response after a punishment.
2) *Instrumental-like reward-focused action selection*: subjects might attempt to recruit an instrumental learning strategy to solve the task, and work primarily towards the reward-associated action (i.e. pressing the ‘reward’ button), treating it as an approach response, while treating the punishment-associated action as the *alternative*, withdrawal response. In this strategy, a punishment-predictive stimulus would signal reward omission, and thus the need to avoid selecting the reward-associated approach action. This reward-focused instrumental learning strategy would be expressed in terms of poorer performance on punishment prediction trials than on reward prediction trials.

The number of data points varied per trial type as a function of performance so errors were transformed into proportional scores (i.e. as a proportion of the total number of [reward or punishment] switch or non-switch trials depending upon the variable being examined). These proportions were then arcsine transformed (2 × arcsine(√*x*)) as is appropriate when the variance is proportional to the mean (Howell, 2002).

In addition to the error rate analysis, we also examined reaction times (RTs; milliseconds) for non-switch trials, switch trials, perseverative trials stratified by both *preceding* outcome and *to be predicted* outcome, response alternation trials and response repetition trials.

## Functional neuroimaging

### Image acquisition

A Siemens TIM Trio 3 T scanner (Magnetom Trio, Siemens Medical Systems, Erlangen, Germany) was used to acquire structural and gradient-echo echo-planar functional images (repetition time = 2000 ms; echo time = 30 ms; 32 axial oblique slices; matrix size dimensions 64 × 64 × 32; voxel size 3 × 3 × 3.75; flip angle 78; slice acquisition orientation axial oblique T > C-24.6; field of view: 192 mm; 6 sessions each of 312 volume acquisitions). The first 12 volumes from each session were discarded to avoid T1 equilibrium effects. In addition, an MPRAGE anatomical reference image of the whole brain was acquired for each subject (repetition time = 2300 ms; echo time = 2.98 ms; 176 slices; matrix size dimensions 240 × 256 × 176, voxel size 1 × 1 × 1) for spatial coregistration and normalization.

### Image analysis

Images were pre-processed and analyzed using SPM5 (Statistical Parametric Mapping; Wellcome Department of Cognitive Neurology, London, UK). Preprocessing consisted of within-subject realignment, coregistration, segmentation, spatial normalization and spatial smoothing. Functional scans were coregistered to the MPRAGE structural image, which was processed using a unified segmentation procedure combining segmentation, bias correction and spatial normalization (Ashburner and Friston, 2005); the same normalization parameters were then used to normalize the EPI images. Finally the EPI images were smoothed with a Gaussian kernel of 8 mm full width half maximum. The canonical hemodynamic response function and its temporal derivative were used as covariates in a general linear model. The parameter estimates, derived from the mean least-squares fit of the model to the data, reflect the strength of covariance between the data and the canonical response functions for given event-types (specified below) at each voxel. Individuals' contrast images were taken to second-level group analyses, in which *t* values were calculated for each voxel, treating inter-subject variability as a random effect. Movement parameters were included as covariates of no interest.

We estimated a general linear model, for which parameter estimates were generated at the onsets of the expected and unexpected reward and punishment outcomes (with zero duration), which co-occurred with the response. An unexpected outcome was the first outcome of a new stage, presented after learning criterion had been obtained, i.e. the outcome signaling contingency reversal. All other outcomes were coded as expected outcomes. There were four trial-types (unexpected reward, unexpected punishment, expected reward, expected punishment) and a two by two factorial design was employed with valence and expectedness as within-subject factors. All four trial types were brought to the second level and whole brain effects were determined by contrasts between these factors at the second level. Reward-based reversal activity was determined by the 1 0 −1 0 (UR UP ER EP) contrast; punishment-based reversal activity was determined by the 0 1 0 −1 contrast; the valence-nonspecific signal was determined by the 1 1 −1 −1 contrast; the valence-specific signal was determined by the 1 −1 −1 1 contrast.

For whole-brain analyses, statistical inference was performed at the voxel level, correcting for multiple comparisons using the family-wise error (FWE) over the whole brain (*P*_WB_FWE_ < 0.05). For illustrative purposes we then extracted the betas from peak voxels using the MarsBar software (Brett et al., 2002).

## Results

### Behavioral

Mean RTs and error rates are presented in Table 1. Each subject completed a mean of 107 (SD 18) reversals, of which 54 (SD 9) were signaled by unexpected reward and 54 (SD 9) by unexpected punishment. Error rates did not differ between reward prediction and punishment prediction trials (*t*_15_ = −0.38, *P =* 0.7). However, subjects were significantly faster to press the reward button than the punishment button across all trials (*t*_15_ = −3.12, *P =* 0.007).

Subjects made significantly more switch errors than non-switch errors (*F*_1,15_ *=* 2378, *P* < 0.001). There was no effect of the valence of the *preceding* (*F*_1,15_ = 0.24, *P* = 0.6) or the *to be predicted* (*F*_1,15_ = 0.23, *P* = 0.64) outcome on switch error rates and there was no interaction between the valence of the *preceding* and the *to be predicted* outcome (*F*_1,15_ = 0.34, *P* = 0.55). For RTs, there was an interaction between the *preceding* and *to be predicted* outcomes (*F*_1,15_ = 7.1, *P* = 0.018), which was driven by subjects being significantly faster to predict reward after unexpected reward, relative to both predicting punishment after unexpected reward (*F*_1,15_ = 7.2, *P* = 0.017) and relative to predicting reward after unexpected punishment (*F*_1,15_ *=* 17.1, *P* = 0.001).

Subjects made an average of 11.7 (SD 3.6) response repetition errors and 12.6 (SD 3.9) response alternation errors, but there was no effect of the preceding outcome on response repetition error rates (*t*_15_ = −0.72, *P =* 0.48) or response alternation error rates (*t*_15_ = −0.13, *P =* 0.90). Thus subjects were not more likely to repeat responses after unexpected reward or switch responses after unexpected punishment. There was no difference in terms of reaction times between response repetition and response alternation trials (*t*_15_ = −1.1, *P =* 0.27).

**Fig. 2:**
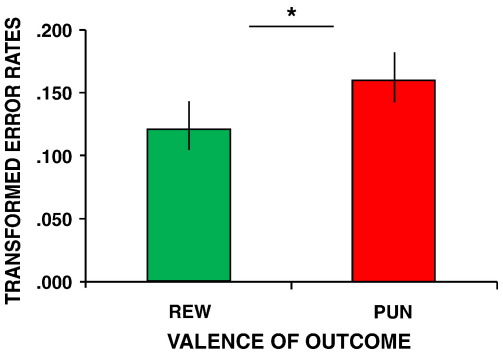
Behavioral data. Perseverative error rates (percentage) following a reversal stratified by the *to be predicted* outcome. *P < 0.05. Error bars represent SEM.

Subjects made an average of 5.6 (SD 3.4) perseverative errors, which did not differ as a function of the preceding outcome (main effect of *preceding*; *F*_1,15_ = 0.41, *P* = 0.53). However, perseverative errors were increased when subjects had to predict punishment relative to reward after any unexpected feedback (main effect of *predicted*; *F*_1,15_ = 4.9, *P* = 0.04; Fig. 2). There was no interaction between *preceding* and *predicted* outcome on perseverative errors (*F*_1,15_ *=* 1.5, *P* = 0.24) and the speed of perseverative responses was equal across trials (*preceding* [*F*_1,15_ *=* 2.2, *P* = 0.20]; *to be predicted* [*F*_1,15_ *=* 1.8, *P* = 0.24]; *interaction* [*F*_1,15_ = 0.34, *P* = 0.59]).

In summary, subjects responded more slowly and exhibited increased perseveration on punishment prediction trials than on reward prediction trials. These results suggest that subjects might have adopted an instrumental-like reward focused action selection strategy (see Materials and methods). In particular, subjects may have evaluated whether to make a GO response to the reward button. If the evidence for reward was not above a certain threshold, then subjects may have withheld responses (NOGO) and made a delayed *alternate* response (i.e. pressed the punishment button). Furthermore the absence of a difference in reward and punishment repetition and alternation error rates suggests that subjects did not recruit a win-stay, lose-shift strategy.

### Functional imaging data

**Fig. 3:**
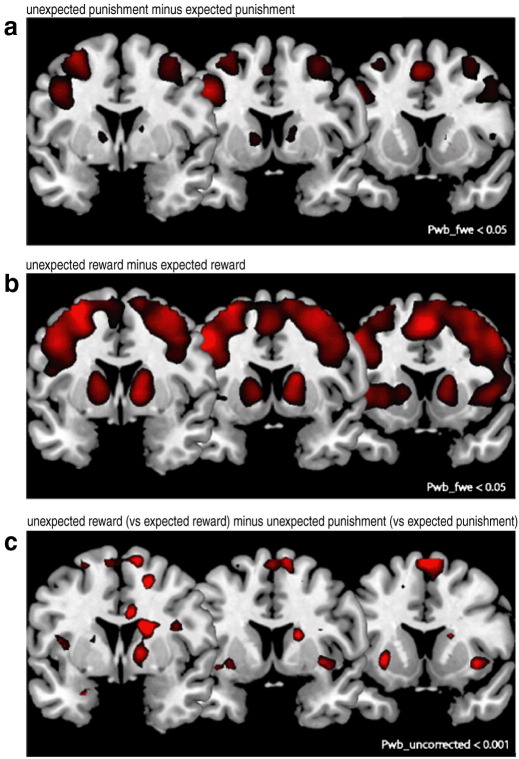
significant whole brain BOLD signals during (a) punishment-based reversals, (b) reward based-reversals and (c) reward-based reversals minus punishment-based reversals.

Data are shown in Fig. 3 and Table 2, Table 3. First, we assessed BOLD responses during the unexpected punishment events that signaled reversal (relative to the expected punishment events). This effect of punishment-based reversal was highly significant in a widespread network of regions, including the striatum, ventrolateral prefrontal cortex, dorsolateral prefrontal cortex, dorsomedial prefrontal cortex and posterior parietal cortex (Table 2; Fig. 3a). The network closely resembled that observed previously during instrumental reversal learning (Cools et al., 2002) (O'Doherty et al., 2001, Cools et al., 2002, O'Doherty et al., 2003, O'Doherty et al., 2004, Remijnse et al., 2005, Hampton & O'Doherty, 2007). Small volume analysis of the caudate nucleus and the putamen revealed that peaks were centered on the anterior ventral striatum (caudate nucleus: Talairach coordinates *x*, *y*, *z =* 12, 9, 4; *T* = 6.1, *P*_SV_FWE_ = 0 < 0.001 and *x*, *y*, *z* = −12, 9, 4; *T* = 5.8, *P*_SV_FWE_ < 0.001; putamen: *x*, *y*, *z* = −15, 9, 4; *T* = 5.6, *P*_SV_FWE_ < 0.001).

Next we assessed BOLD responses during the unexpected reward events that signaled reversal (relative to the expected reward events). This contrast revealed a very similar network of regions to the unexpected punishment contrast (Table 3; Fig. 3b). However, the pattern of activity was more extensive. Furthermore small volume analyses of the caudate nucleus and the putamen revealed that peaks were centered on more dorsal and posterior parts of the striatum (caudate nucleus: *x*, *y*, *z =* 18, 9, 15, *T* = 9.2, *P*_SV_FWE_ < 0.001 and *x*, *y*, *z* = −18, 3, 19, *T* = 7.7, *P*_SV_FWE_ < 0.001; putamen: *x*, *y*, *z* = −18, 0 .8, *T* = 7.5, *P*_SV_FWE_ < 0.001).

To further investigate these differences within the striatum, we also computed a direct contrast representing the difference between reward- and punishment-based reversal learning, i.e. the interaction between expectedness and valence. Consistent with the above analyses, this contrast revealed greater activity during unexpected reward (*relative to* expected reward) than during unexpected punishment (*relative to* expected punishment) in the more posterior and dorsal parts of the striatum, even at the whole-brain level (*x*, *y*, *z =* 18, −6, 8, *T* = 5.4, *P*_WB_FWE_ = 0.02), but in no other regions. Small volume analyses confirmed this observation and revealed peaks in the posterior and dorsal part of the bilateral caudate nucleus at *x*, *y*, *z =* 21, −3, 22 (*T* = 5.2, *P*_SV_FWE_ = 0.001) and *x*, *y*, *z* = −12, −9, 15 (*T* = 3.9, *P*_SV_FWE_ = 0.02) as well as in the dorsolateral part of the bilateral putamen at *x*, *y*, *z* = −21, −6, 8 (*T* = 4.1, *P*_SV_FWE_ = 0.01) and 24, −6, 8 (*T* = 4.0, *P*_SV_FWE_ = 0.02).

**Fig. 4:**
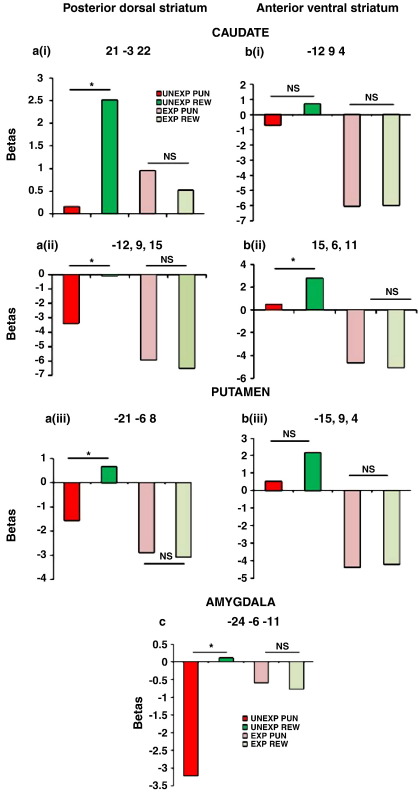
(a) Parameter estimates (betas) extracted for the four trial-types (unexpected punishment, unexpected reward, expected punishment and expected reward) from striatal peak voxels revealed by the contrast map reflecting the valence by expectedness interaction (unexpected reward relative to expected reward minus unexpected punishment relative to expected punishment). (b) Parameter estimates (betas) extracted for the four trial-types from striatal peak voxels revealed by the contrast map reflecting the main effect of expectedness (both unexpected outcomes minus both expected outcomes). More posterior and dorsal regions of striatum activate solely for reward, while anterior and ventral regions are activated by both reward and punishment. (c) Parameter estimates extracted for the four trial-types from amygdala peak voxels from the interaction map**.** Numbers represent the Talairach voxel coordinates of the extracted peaks. Note that the selection of these data was not independent from the whole-brain analyses; data are plotted for illustration purposes only. Furthermore although we plot each trial type separately, only the differences between trial types should be interpreted.

These analyses suggest that different parts of the striatum exhibit distinct responses to punishment, with the ventral and anterior striatum responding to both unexpected reward and unexpected punishment, in a valence-nonspecific fashion, but with the more dorsal and posterior striatum responding to unexpected reward in a valence-specific fashion. For illustration purposes, we have plotted, in Fig. 4 signal change extracted from these striatal peaks of reward-signed activity (Fig. 4a), as well as that from striatal peaks of unsigned activity (Fig. 4b) [the latter obtained from a small volume analysis within the striatum of a fourth contrast representing the main effect of expectedness, i.e. differences between both types of unexpected outcomes and both types of expected outcomes (caudate nucleus: *x*, *y*, *z =* 15, 6 11, *T* = 8.7, *P*_SV_FWE_ < 0.001 and *x*, *y*, *z* = −12, 9, 4, *T* = 7.9, *P*_SV_FWE_ < 0.001; putamen: *x*, *y*, *z* = −15, 9, 4, *T* = 7.6, *P*_SV_FWE_ < 0.001)]. Fig. 4 shows that the more anterior and ventral part of the striatum exhibits BOLD responses that are largely equally enhanced during unexpected reward and unexpected punishment. Conversely, the more posterior, dorsal and lateral parts of the striatum exhibit BOLD responses that are increased for unexpected reward, but not for unexpected punishment. There were no BOLD responses that were greater for unexpected punishment than for unexpected reward.

Finally, given emphasis in prior literature on the amygdala in punishment processing, we also performed supplementary small volume analysis of the anatomically defined amygdala. This analysis revealed significantly greater activity during unexpected reward (*relative to* expected reward) than during unexpected punishment (*relative to* expected punishment) (Fig. 3c; expectedness × valence interaction in *x*, *y*, *z* = −24, 0, −26, *T* = 3.7, *P*_SV_FWE_ = 0.01 and *x*, *y*, *z* = −24, −6, −11, *T* = 3.3, *P*_SV_FWE_ = 0.03). Extraction of data from these peaks revealed that this interaction was due to reduced activity during unexpected punishment, rather than due to increased activity during unexpected reward (Fig. 4c). Indeed small volume analyses of the amygdala in the contrasts subtracting unexpected outcomes from expected outcomes revealed that BOLD responses were below baseline only for unexpected punishment (right: *x*, *y*, *z* = −24, −9, −15, *T* = 5.4, *P*_SV_FWE_ < 0.001; *x*, *y*, *z* = and left: *x*, *y*, *z* = 27, −9, −15, *T* = 4.1, *P*_SV_FWE_ = 0.004 and *x*, *y*, *z* = 24, −3, −22, *T* = 3.5, *P*_SV_FWE_ = 0.02), but not for unexpected reward (no supra-threshold clusters).

## Discussion

The present results reveal distinct responses to punishment in the anterior ventral and posterior dorsal/lateral striatum. Specifically, a subcortical region extending into the anterior ventral striatum exhibited BOLD responses during both unexpected reward and unexpected punishment whereas more posterior dorsal/lateral regions of the striatum exhibited BOLD responses only during unexpected reward. These data demonstrate that distinct parts of the striatum exhibit dissociable responses to punishment during reversal learning and provide a means of reconciling a number of previously disparate studies.

The regional distribution of activity in the anterior ventral and posterior dorsal/lateral parts of the striatum is reminiscent of known anatomical subdivisions of the striatum (Haber et al., 2000, Voorn et al., 2004), which have been associated with motivational versus cognitive/motor aspects of behavior respectively. Specifically, whereas the ventral striatum has been implicated in the prediction of biologically relevant events, such as rewards and punishments, in Pavlovian paradigms, the dorsal striatum has been implicated in the instrumental control of actions based on these predictions (Montague et al., 1996) (Yin et al., 2006). For example, O'Doherty et al. (2004) have revealed dissociable reward prediction error responses in the ventral and dorsal striatum during Pavlovian and instrumental conditioning respectively. Thus the ventral striatum might be primarily concerned with Pavlovian *states*, whereas the dorsal striatum might participate in reinforcing instrumental *actions* that would act to improve the predicted state. Consistent with this hypothesis, the pattern of activity in the anterior ventral striatum was more pronounced than that in the posterior dorsal/lateral striatum during our Pavlovian task, which required the prediction of rewards and punishment. Whereas activity in the ventral striatum in the study by O'Doherty et al. (2004) was reward-signed, our study revealed that this anterior ventral signal was mostly valence-nonspecific (Fig. 4b). Thus activity in this anterior ventral region was above average not only for unexpected reward but also for unexpected punishment. While the study by O'Doherty et al. (2004) did not measure punishment-related activity, similar valence-nonspecific prediction error signals have been observed previously, albeit only in studies in which Pavlovian tasks were employed (Seymour et al., 2005, Seymour et al., 2007a, Jensen et al., 2007, Delgado et al., 2008). This pattern might reflect overlapping and/or inter-mingled representations of appetitive and aversive signals, as was the case in a recent study by Seymour et al., 2007a, Seymour et al., 2007b. The alternative hypothesis is that it reflects a single valence-independent signal, for example reflecting a mismatch between expected and actual outcomes (Redgrave et al., 1999, Zink et al., 2003; Jensen et al., 2007, Matsumoto & Hikosaka, 2009). However results from our previous pharmacological studies with this task (Cools et al., 2006, Cools et al., 2009) have revealed valence-dependent drug effects, and dependency of the relative ability to learn from reward or punishment on striatal dopamine levels (Cools et al., 2009). Specifically, the effect of dopaminergic manipulation was restricted to the unexpected punishment condition and we anticipate that this dopaminergic effect is accompanied by modulation of striatal signal change related to unexpected punishment. Such striatal signal change related to unexpected punishment was observed in the present study, but only in the ventral anterior part of the striatum. Therefore the valence-specificity of the previously observed behavioral effect suggests that the punishment-related signal change observed here is also valence-specific, but that it overlaps with the reward-related signal change. We are currently testing this hypothesis by examining the effects of dopaminergic manipulations on striatal signal change.

In addition to the anterior ventral valence-nonspecific effect, we also observed a valence-specific signal in more posterior and dorsal/lateral parts of the striatum, which has been associated with outcome-guided action selection rather than outcome prediction. The behavioral performance pattern observed suggests that, in addition to the prediction mechanism, subjects might have additionally recruited an instrumental mechanism, which is likely driven primarily by a positive, reward-signed signal associated with the state in which punishment is not expected (i.e. (Dayan and Seymour, 2008)). In the present task, subjects were much faster to repeat reward responses following unexpected reward and made considerably more perseverative errors following unexpected feedback when they had to predict punishment. These behavioral findings support the proposition that subjects tended to work towards the reward-associated action (i.e. pressing the ‘reward’ button), perhaps treating it as a GO response. In this strategy, a punishment-predictive stimulus would signal reward omission, and thus the need to avoid selecting that reward-associated GO action. The hypothesis that subjects treated the punishment-associated action as a NOGO response is consistent with the increased RTs on these trials.

Our data reconcile a variety of prior, apparently contradictory, studies. Specifically, some studies have shown an increase in striatal activity during punishment in studies of Pavlovian aversive conditioning (Seymour et al., 2005, Jensen et al., 2007, Menon et al., 2007), while other studies have demonstrated decreased activity during punishment after avoidance failure in studies of instrumental aversive conditioning (Kim et al., 2006, Pessiglione et al., 2006, Yacubian et al., 2006). Unlike these prior studies, the present study demonstrates *simultaneous* reward-signed activity in the posterior dorsal/lateral striatum and absolute reward- *and* punishment-related activity in the anterior ventral striatum. These distinct activity patterns may reflect the simultaneous recruitment of Pavlovian prediction and instrumental action selection mechanisms. Distinct recruitment of one or both of these mechanisms by distinct behavioral tasks may explain the discrepancies across previous studies. That said, it should be noted that the recruitment of distinct mechanisms was not controlled experimentally in our paradigm and the design of the present task differs from that of these prior tasks in a number of ways. Accordingly, caution should be exercised when extrapolating across studies. In future studies, punishment-related signal change in the striatum should be compared directly using Pavlovian and instrumental paradigms to assess the speculation that controllable (instrumental) punishment and uncontrollable punishment implicate distinct parts of the striatum.

Reward-signed signal change was also observed in the amygdala, although the pattern in the amygdala was qualitatively different from that seen in the posterior dorsal/lateral striatum. Specifically, activity in the amygdala decreased below baseline during unexpected punishment rather than increasing above baseline during unexpected reward. Together our pattern of unsigned activity in the ventral striatum and reward-signed activity in the amygdala matches that found previously (Seymour et al., 2005) and further highlights the role of the amygdala in appetitive processing.

Our findings have implications for understanding the mechanisms underlying the dopaminergic modulation of striatal BOLD responses seen in previous studies of reversal learning, which likely involve both Pavlovian and instrumental control mechanisms. In these prior studies, striatal activity, and the effect of dopamine during the punishment event that led to reversal, was centered on its anterior ventral rather than its posterior dorsal parts. Together with these prior results, the present data suggest that the striatal activity (and its reduction by dopamine) during punishment-driven reversal learning could be driven by (disruption of) processing associated with punishment, rather than a modulation of reward-related processing such as reward anticipation or reward-focused learning (see Introduction). However a pharmacological fMRI study with this paradigm which is presently underway will clarify this. Unlike our pharmacological fMRI studies of reversal learning (Cools et al., 2007, Dodds et al., 2008), some recent studies of learning have failed to observe modulation by dopamine or Parkinson's disease of processing associated with punishment, or with the negative reward prediction error (Pessiglione et al., 2006, Schonberg et al., 2009, Rutledge et al., 09). Performance on these tasks might depend to different degrees on Pavlovian and/or instrumental control mechanisms. Although other neurotransmitters, like serotonin, should be considered as plausible candidates ((Daw et al., 2002, Cools et al., 2008), it is possible that punishment-related activity in the striatum during reversal learning reflects dopaminergic influences from the midbrain. The current paradigm opens avenues for assessing the degree to which known opposite effects of dopaminergic drugs on punishment- and reward-based reversal learning (Cools et al., 2006, Cools et al., 2009) are accompanied by opposite effects on punishment- and reward-related activity in the anterior ventromedial striatum.

## Figures and Tables

**Table 1:** Behavioral data. Error rates (percentage) and reaction times (ms) (standard error of the mean) for both switch and non-switch trials. Switch trials are stratified by the valence of the unexpected feedback that signaled the switch (*preceding* outcome), and by the valence of the feedback that should have been predicted (*to be predicted* outcome). The switch trial is the trial immediately after the unexpected feedback, perseverative errors are all consecutive errors after this switch trial.

*A: Non-switch trials*		
	Reward	Punishment
RT	765 (19)	788 (19)
Errors	0.08 (0.86)	0.08 (0.61)

*B: Switch trials*				
	Unexpected reward		Unexpected punishment	
	Reward	Punishment	Reward	Punishment
*Reaction times*				
Switch	690 (27)	772 (36)	807 (32)	759 (31)
Perseveration	993 (110)	1071 (64)	886 (61)	937 (48)

*Error rates*				
Switch	11.15 (1.01)	12.37 (1.11)	12.44 (0.91)	11.87 (0.90)
Perseveration	2.01 (0.61)	3.54 (0.76)	2.65 (0.49)	3.44 (0.72)

**Table 2:** Unexpected punishment minus expected punishment.

Label	Talairach coordinates (*x*, *y*, *z*) of peak locus	*T*	Cluster size (*K*)
Left posterior parietal cortex	−42 −48 −49	10.7	541
Right posterior parietal cortex	39 −54 45	9.4	432
Dorsomedial prefrontal cortex	0 21 49	8.8	217
Left frontal eye fields, extending into left dorsolateral prefrontal cortex	−45 9 38	9.5	498
Right dorsolateral prefrontal cortex	45 30 34	8.6	433
Left ventrolateral prefrontal cortex/insula	−30 21 −4	6.8	74
Right ventrolateral prefrontal cortex/insula	36 24 −4	7.7	148
Left lateral anterior prefrontal cortex	−39 54 11	6.2	22
Right lateral anterior prefrontal cortex	33 51 4	5.5	6
Right lateral orbitofrontal cortex	33 54 −8	5.4	7
Left anterior striatum	−12 6 4	6.3	20
Right anterior striatum	12 9 4	5.8	17
Left lateral cerebellum	−33 −63 −30	7.07.0	55
Right lateral cerebellum	30 −63 −30	8.6	57
Left medial cerebellum	−9 −78 −30	6.2	19
Right medial cerebellum	9 −75 −76	5.6	11
Right posterior temporal cortex	48 −27 −8	6.9	57

Local maxima of suprathreshold clusters at *P*_WB_FWE_ < 0.05.

**Table 3:** Unexpected reward minus expected reward.

Label	Talairach coordinates (*x*, *y*, *z*) of peak locus	*T*	Cluster size (*K*)
Left posterior parietal cortex, extending into right posterior parietal cortex	−42 −48 −49	13.7	741
Right ventrolateral prefrontal cortex/insula, extending into left ventrolateral prefrontal cortex/insula, bilateral dorsolateral prefrontal cortex, lateral anterior and orbital frontal cortex, dorsomedial prefrontal cortex, striatum, and thalamus	36 21 −4	11.3	5041
Left cerebellum, extending into right cerebellum	−30 −13 −30	10.9	702
Posterior cingulate cortex	3 −30 26	5.9	28
Right posterior temporal cortex	60 −36 −8	8.2	167
Left posterior temporal cortex	−60 −36 −8	5.5	28
Midbrain	3 −27 −22	4.9	7

Local maxima of suprathreshold clusters at *P*_WB_FWE_ < 0.05.

## Appendix A Supplementary data

Fig. S1 Data from peak striatal voxels estimated using a finite impulse response model. Values represent percentage signal change. This figure can be compared to Fig. 4 in the main manuscript.
