# Supplementary material for: Dissociable responses to punishment in distinct striatal regions during reversal learning
Source: Neuroimage. 2010 Jul 15;51(4-4):1459–67. doi: 10.1016/j.neuroimage.2010.03.036 (PMC3038262; doi:10.1016/j.neuroimage.2010.03.036)

**SUPPLEMENTARY METHODS**

Finite impulse response (FIR) event time courses were extracted using the marsbar toolbox for SPM from the peak voxels identified in the main text.

**SUPPLEMENTARY FIGURE1**

Data from peak striatal voxels estimated using a finite impulse response model. Values represent percentage signal change. This figure can be compared to figure 4 in the main manuscript


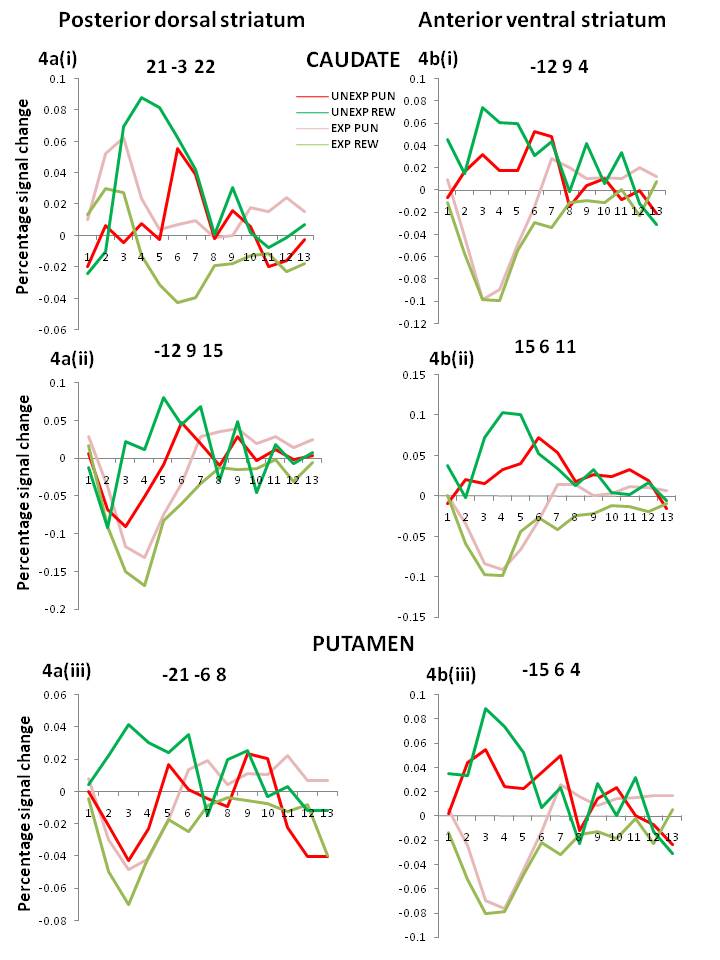

Supplement: Fig. S1 — Data from peak striatal voxels estimated using a finite impulse response model. Values represent percentage signal change. This figure can be compared to Fig. 4 in the main manuscript. [file mmc1.doc]
